# COX-2及其转录因子NFAT3和c-Fos在非小细胞肺癌中的表达及意义

**DOI:** 10.3779/j.issn.1009-3419.2010.11.07

**Published:** 2010-11-20

**Authors:** 晓鸿 赵, 照丽 陈, 守华 赵, 捷 赫

**Affiliations:** 1 100021 北京，北京协和医学院，中国医学科学院，肿瘤医院肿瘤研究所胸外科 Department of Thoracic Surgery, Cancer Institute and Hospital, Chinese Academy of Medical Sciences, Peking Union Medical College, Beijing 100021, China; 2 277500 滕州，山东省滕州市中心人民医院胸外科 Department of Thoracic Surgery, Tengzhou Center People Hospital, Tengzhou 277500, China

**Keywords:** 肺肿瘤, 环氧化酶-2, 活性T细胞核因子, 原癌基因*c-fos*编码产物, Lung neoplasms, Cyclooxygenase 2, NFATC transcription factors, Proto-oncogene proteins c-fos

## Abstract

**背景与目的:**

环氧化酶2（cyclooxygenase 2, COX-2）是前列腺素合成过程的关键酶，在肿瘤的发生发展中发挥重要作用。COX-2的表达受多种转录因子的调节。

**方法:**

本研究利用含有159例非小细胞肺癌的组织芯片，通过免疫组化检测COX-2、原癌基因*c-fos*编码产物（c-Fos蛋白）、原癌基因*c-jun*编码产物（c-Jun蛋白）以及活性T细胞核因子3（nuclear factor of activated T cells 3, NFAT3）的表达情况，分析其表达之间的关系及其与临床病理因素之间的相关性。

**结果:**

159例肺癌标本中COX-2阳性表达率为42.8%，在鳞癌中阳性表达率高于腺癌（52.9% *vs* 31.3%, *χ*^2^=7.723, *P*=0.005）。COX-2的表达与分化程度相关，分化越好表达水平越高（*χ*^2^=7.600, *P*=0.022）。159例肺癌标本中，COX-2与c-Fos的表达具有相关性（*r*=0.456, *P* < 0.001），COX-2与NFAT3的表达具有相关性（*r*=0.294, *P* < 0.001），NFAT3与c-Fos表达之间也具有相关性（*r*=0.231, *P*=0.003）。

**结论:**

在非小细胞肺癌组织中COX-2的表达与转录因子NFAT3和c-Fos的表达明显相关。

## Abstract (en)

**Background and objective:**

Cyclooxygenases (COX), the key enzymes in the conversion of arachidonic acid (AA) to prostaglandins (PGs), are involved in initiation and progression of cancer. The aim of this study is to explore the relationship between the expressions of COX-2 and several transcription factors in non-small cell lung cancer.

**Methods:**

Immunohistochemistry was performed to assay the expression levels of COX-2, c-Fos, c-Jun and nuclear factor of activated T cells 3 (NFAT3) in tissue microarray containing 159 tumor tissues of non-small cell lung cancer.

**Results:**

The positive rate of COX-2 expression was 42.8%, and the expression of COX-2 was significantly higher in squamous cell carcinoma than that in adenocarcinoma (52.9% *vs* 31.3%, *χ*^2^=7.723, *P*=0.005). The expression of COX-2 was significantly associated with differentiation grade, with the lower level in the poorer differentiation grade group (*χ*^2^=7.600, *P*=0.022). In this panel of samples, the expression of COX-2 was significantly correlated with c-Fos expression (*r*=0.456, *P* < 0.001) and NFAT3 level (*r*=0.294, *P* < 0.001). The correlation between the expressions of NFAT3 and c-Fos were also observed (*r*=0.231, *P*=0.003).

**Conclusion:**

The expression of COX-2 was significantly associated with the expressions of transcription factors NFAT3 and c-Fos in nonsmall cell lung cancer.

环氧化酶2（cyclooxygenase 2, COX-2）是前列腺素合成过程中的关键酶，与肿瘤的发生发展具有密切的关系。在很多恶性肿瘤组织中均可检测到COX-2的过表达^[1-5]^。研究表明一些细胞因子、生长因子、佛波酯、化学致癌物以及癌基因均能够诱导COX-2的表达^[6-9]^。COX-2特异抑制剂能够有效抑制肿瘤的生长^[10]^。COX-2的表达水平受多种机制的调节。NF-κB等多种转录因子能调节COX-2基因的转录活性^[11]^；也有研究^[12]^证实COX-2 mRNA的稳定性受TGF-β和Ras协同作用的调节。

活性T细胞核因子（nuclear factor of activated T cells, NFAT）是细胞内普遍存在的一类转录因子家族。该家族包括5个成员，在多数情况下与活化蛋白质1（activator protein 1, AP-1）等其它转录因子结合成异二聚体，与特定的DNA序列结合，启动下游基因的转录。NFAT在多种肿瘤细胞中调节细胞增殖、凋亡、转移等活性。研究^[13, 14]^表明，在结肠癌和乳腺癌细胞中NFAT和AP-1共同作用可以调节COX-2的转录。在T细胞、肾小球上皮、神经胶质细胞中NFAT也是COX-2的转录因子^[15-17]^。亚砷酸盐引起支气管上皮细胞恶变的过程中，NFAT通过激活COX-2的转录发挥了关键作用^[18]^。

已有的研究表明NFAT和COX-2在非小细胞肺癌中均过表达^[19, 20]^，但NFAT与COX-2之间的关系未见报导。本课题组前期对NFAT家族的研究表明，NFAT1、NFAT2、NFAT4与COX-2的表达均无相关性（尚未发表）。为进一步探讨肺癌中调节COX-2表达的转录因子，本研究在非小细胞肺癌组织中检测COX-2及其可能的转录调节因子NFAT3、AP-1的亚基c-Fos蛋白和c-Jun蛋白的表达水平，分析其表达之间的关系及临床意义。

## 1 材料与方法

### 1.1 临床资料

回顾性收集1998年1月-2001年12月间在中国医学科学院肿瘤医院接受手术治疗的原发性非小细胞肺癌病例159例，均未进行术前放疗和化疗。所有159例病例均具有详细的临床病理资料和5年随访资料。其中男性111例，女性48例；腺癌74例，鳞癌85例；高分化8例，中分化103例，低分化48例；Ⅰ期70例，Ⅱ期和Ⅲ期89例。

### 1.2 试剂与方法

所有石蜡标本的HE染色切片均经病理医生阅片确诊，每例患者分别标记两个肺癌组织点以及两个距癌5 cm以远的正常肺组织点，制备组织芯片。组织芯片上，每个组织点直径1 mm，每个病例包括4个点。连续切片厚度4 μm。兔抗人COX-2多抗（RB-9072-P，工作浓度1:200）和兔抗人c-Fos多抗（RB-9431-P，工作浓度1:250）购自美国Thermo Fisher Scientific公司，兔抗人c-Jun多抗购自美国SAB公司（Ab-243, 1:200），兔抗人NFAT3的抗体购自英国Abcam公司（ab3447，工作浓度1:50）。PV9000及DAB显色液购自北京中杉金桥生物技术有限公司。切片经二甲苯脱蜡，梯度乙醇水化。微波炉中高火5 min、中低火15 min进行抗原热修复，3%双氧水、80%甲醇室温30 min消除内源性过氧化酶活性。一抗4 ℃孵育过夜，PBS漂洗，PV9000室温孵育，DAB显色，苏木素复染，1%盐酸、75%乙醇分化，1%氨水返蓝，过梯度乙醇脱水，封片。PBS代替一抗为阴性对照。

### 1.3 免疫组化结果判断

结果由中国医学科学院肿瘤医院两位病理医生分别独立读片。c-Fos和c-Jun核染色为阳性，COX-2浆染色为阳性，NFAT3核、浆染色均计为阳性。免疫组化结果均根据细胞染色强度评分0、1、2、3和4；根据阳性细胞的百分数评分0（≤5%）、1（6%-25%）、2（26%-50%）、3（51%-75%）、4（76%-100%）。计算芯片上每个组织点染色强度和阳性细胞数评分的乘积；根据得分判断0为阴性，≤4分为弱阳性，>4分为强阳性。

### 1.4 统计学方法

采用SPSS 13.0软件进行统计分析。组间表达差异比较应用*χ*^2^检验，蛋白表达相关性分析用*Spearman*等级相关分析。*P* < 0.05为差异有统计学意义。

## 2 结果

### 2.1 COX-2、c-Fos、c-Jun、NFAT3在非小细胞肺癌中的表达情况

在159例非小细胞肺癌组织中，COX-2的阳性表达率为42.8%（68/159）（图 1）。在鳞癌中阳性表达率为52.9%（45/85），高于腺癌中的31.3%（23/74），差异具有统计学意义（*χ*^2^=7.723, *P*=0.005）。COX-2的表达与分化程度相关，高分化组的阳性表达率为87.5%（7/8），中分化组为42.7%（44/103），低分化组为35.4%（17/48），三组之间的差异具有统计学意义（*χ*^2^=7.600, *P*=0.022）。在男性中阳性表达率为47.7%（53/111），高于女性的31.3%（15/48），但差异无统计学意义（*χ*^2^=3.726, *P*=0.054）（表 1）。COX-2表达在不同年龄、吸烟及病理分期的非小细胞肺癌患者之间无差异。而且COX-2的表达与患者的预后无相关性。在159例非小细胞肺癌组织中，c-Fos的阳性表达率为45.9%（73/159），在鳞癌中阳性表达率为52.9%（45/85），高于腺癌中的37.8%（28/74），但差异无统计学意义（*χ*^2^=3.634, *P*=0.057）。c-Fos的表达与患者的年龄、性别、吸烟、分化程度、淋巴结转移和临床分期均无明显相关性（表 1）。c-Jun的阳性表达率仅14.5%（23/159），其表达与临床病理因素无相关性。NFAT3在28.3%（45/159）的肺癌组织中阳性表达，其表达与各种病理因素之间无相关性（表 2）。

**1:**
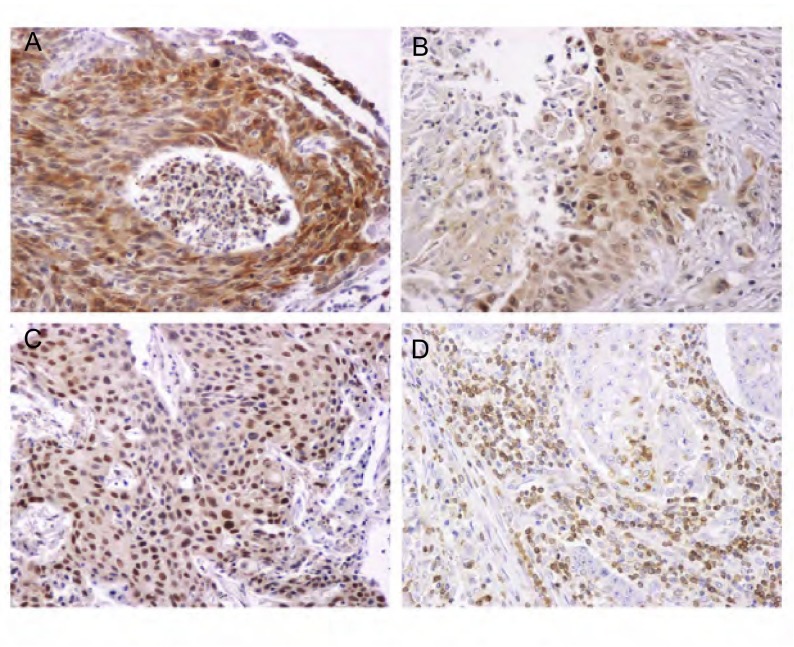
COX-2、c-Fos、c-Jun和NFAT3在非小细胞肺癌组织中的表达（IHC, ×200）。A：COX-2在非小细胞肺癌组织中阳性染色；B：c-Fos在非小细胞肺癌组织中阳性染色；C：c-Jun在非小细胞肺癌组织中阳性染色；D：NFAT3在非小细胞肺癌组织中阳性染色。 Expressions of COX-2, c-Fos, c-Jun and NFAT3 in non-small cell lung cancer (IHC, ×200). A: positive staining of COX-2 in non-small cell lung cancer tissues; B: positive staining of c-Fos in non-small cell lung cancer tissues; C: positive staining of c-Jun in non-small cell lung cancer tissues; C: positive staining of NFAT3 in non-small cell lung cancer tissues.

**1:** COX-2、c-Fos表达与非小细胞肺癌患者临床病理因素之间的关系 Association between expression of COX-2 and c-Fos and clinicopathologic characteristics of non-small cell lung cancer

Clinicopathologic characteristics(*n*=159)	*n*	Expression of COX-2		*X*^2^	*P*	Expression of c-Fos		*X*^2^	*P*
		Positive	Rate			Positive	Rate		
Age				0.931	0.335			0.090	0.764
≤60	63	24	38.1%			28	44.4%		
> 60	96	44	45.8%			45	46.9%		
Gender				3.726	0.054			0.499	0.480
Male	111	53	47.7%			53	47.7%		
Female	48	15	31.3%			20	41.7%		
Smoking status				0.045	0.832			0.006	0.939
Never	67	28	41.8%			31	46.3%		
Smoker	92	40	43.5%			42	45.7%		
Differentiation				7.600	0.022			4.208	0.122
High	8	7	87.5 %			6	75.0%		
Moderate	103	44	42.7%			49	47.6%		
Low	48	17	35.4%			18	37.5%		
Histologic type				7.723	0.005			3.634	0.057
Adenocarcinoma	74	23	31.1%			28	37.8%		
Squamous cell carcinoma	85	45	52.9%			45	52.9%		
Lymph node metastasis				0.800	0.371			0.234	0.628
No	73	34	39.5%			32	43.8%		
Yes	86	34	46.6%			41	47.7%		
TNM staging				0.018	0.895			0.089	0.297
Ⅰ + Ⅱ	109	47	43.1%			47	43.1%		
Ⅲ	50	21	42.0%			26	52.0%		

**2:** c-Jun、NFAT3表达与非小细胞肺癌患者临床病理因素之间的关系 Association between expression of c-Jun and NFAT3 and clinicopathologic characteristics of non-small cell lung cancer

Clinicopathologic characteristics (*n*=159)	*n*	Expression of c-Jun		*X*^2^	*P*	Expression of NFAT3		*X*^2^	*P*
		Positive	Rate			Positive	Rate		
Age				3.595	0.058			0.004	0.951
≤60	63	5	7.9%			18	28.6%		
> 60	96	18	18.8%			27	28.2%		
Gender				0.911	0.340			1.890	0.169
Male	111	18	16.2%			35	31.5%		
Female	48	5	10.4%			10	20.8%		
Smoking status				1.511	0.219			1.196	0.158
Never	67	7	10.2%			15	22.3%		
Smoker	92	16	17.4%			30	32.6%		
Differentiation				2.523	0.283			3.326	0.190
High	8	2	25.0 %			2	25.0%		
Moderate	103	17	16.5%			34	33.0%		
Low	48	4	8.3%			9	18.8%		
Histologic type				2.804	0.094			0.470	0.493
Adenocarcinoma	74	7	9.5%			19	25.7%		
Squamous cell carcinoma	85	16	18.8%			26	30.6%		
Lymph node metastasis				0.425	0.515			0.014	0.904
No	73	12	39.5%			21	28.8%		
Yes	86	11	46.6%			24	27.9%		
TNM staging				0.736	0.391			1.167	0.280
Ⅰ + Ⅱ	109	14	12.8%			28	25.7%		
Ⅲ	50	9	18.0%			17	34.0%		

### 2.2 COX-2与c-Fos、c-Jun、NFAT3表达的相关性（表 3）

**3:** 非小细胞肺癌中COX-2和c-Fos、c-Jun、NFAT3表达强度的相关性 Correlation between the expressions of COX-2 and c-Fos, c-Jun, NFAT3 in non-small cell lung cancer

Expression of COX-2	Expression of c-Fos			*r*	*P*	Expression of c-Jun			*r*	*P*	Expression of NFAT3			*r*	*P*
	Negative	Weak positive	Strong positive			Negative	Weak positive	Strong positive			Negative	Weak positive	Strong positive		
Negative	64	21	6	0.456	< 0.001	85	6	0	0.303	< 0.001	73	14	40	0.294	< 0.001
Weak positive	20	22	3			37	7	1			31	7	7		
Strong positive	2	8	13			14	7	2			10	3	10		

在本研究的159例标本中，COX-2的表达与c-Fos的表达呈正相关（*r*=0.456, *P* < 0.001）。COX-2的表达与c-Jun的表达具有相关性（*r*=0.303, *P* < 0.001）。COX-2的表达与NFAT3的表达呈正相关（*r*=0.294, *P* < 0.001）。

### 2.3 NFAT3与c-Fos、c-Jun表达的相关性（表 4）

**4:** 非小细胞肺癌中NFAT3与c-Fos、c-Jun表达强度的相关性 Correlation between the expressions of NFAT3 and c-Fos or c-Jun in non-small cell lung cancer

Expression of NFAT3	Expression of c-Fos			*r*	*P*	Expression of c-Jun			*r*	*P*
	Negative	Weak positive	Strong positive			Negative	Weak positive	Strong positive		
Negative	69	32	13	0.231	0.003	107	7	0	0.428	< 0.001
Weak positive	12	10	2			20	4	0		
Strong positive	5	9	7			9	9	3		

NFAT3与c-Fos的表达呈正相关（*r*=0.231, *P*=0.003）。NFAT3与c-Jun的表达呈正相关（*r*=0.428, *P* < 0.001）。

## 3 讨论

COX-2是前列腺素合成过程中的关键酶，它的表达受到细胞内多条信号通路的调节。COX-2在多种肿瘤组织中表达上调，而且能够调节肿瘤细胞的凋亡、侵袭能力，调节肿瘤血管形成和肿瘤微环境中炎症因子的形成。多项研究证实，COX-2的表达与肺癌的发生相关。Hosomi等^[21]^研究表明，在71.6%肺不典型腺瘤样增生组织中COX-2呈阳性表达。Mascaux等^[22]^研究也表明，在肺的重度不典型增生和原位癌组织中，COX-2的表达显著高于正常组织和轻中度不典型增生。本研究结果表明，随着分化程度的增高COX-2的表达水平增加。Wolff等^[23]^研究也发现COX-2在分化好的肺癌中的表达高于分化差的肺癌。另外，本研究检测的159例非小细胞肺癌组织中，COX-2在鳞癌中的阳性表达率高于腺癌。已有的几项研究^[19, 23, 24]^发现COX-2在腺癌中的阳性表达率偏高。在利用中国人肺癌组织标本的研究中，COX-2在肺鳞癌和腺癌中的表达无统计学差异^[25]^，这可能是样本来源不同造成的差异。另外，COX-2的表达与非小细胞肺癌患者的预后无明显相关^[26]^；在本研究中也未发现这种相关性，这表明COX-2主要参与肺癌的发生和早期阶段。

NFAT能够与转录因子AP-1相互作用激活基因的转录。在*COX-2*基因的启动子区NFAT的结合位点与AP-1的结合位点相邻，而且是COX-2转录激活必需的转录因子^[15, 27]^。Wolfe等^[28]^解析出了NFAT与AP-1相互作用的晶体结构和关键氨基酸残基。NFAT蛋白由3个结构域构成：NHR（NFAT同源结构域）、RHR（Rel同源结构域）和C端结构域。其中，RHR结构域的功能是与DNA结合、与其它转录因子相互作用。NFAT亚型之间RHR结构域高度同源，都能与相同的DNA序列结合^[29]^。不同的细胞中，调节COX-2转录的NFAT亚型不同。在乳腺癌细胞中，NFAT1和AP-1共同激活了COX-2的表达，增加了乳腺癌细胞的侵袭能力^[14]^。结肠癌细胞中NFAT2和AP-1共同作用激活了COX-2的转录，增强细胞的运动能力^[13]^。Huang等^[18]^研究表明，在正常肺支气管上皮细胞中，致癌剂亚砷酸盐引起的抗凋亡作用是通过激活NFAT3的转录活性而提高COX-2的表达水平来实现的。本研究的结果也表明，在非小细胞肺癌中，NFAT3的表达与COX-2的水平具有明显相关性。本实验室前期研究发现，虽然NFAT1和NFAT4阳性表达率高于NFAT3，但与COX-2的表达水平之间无相关性；可能通过调节其它基因的转录影响肺癌的发生发展。虽然NFAT2的表达与COX-2水平具有相关性，但NFAT2的阳性表达率很低，仅为11%，在肺癌中可能不发挥重要作用（待发表）。这些结果说明，非小细胞肺癌中不同的NFAT亚型可能发挥不同的作用，COX-2的表达主要受NFAT3的调节。

AP-1由Fos（c-Fos、Fos-B、Fra-1和Fra-2）和Jun（c- Jun、Jun-B和Jun-D）蛋白家族成员组成。Jun蛋白家族成员必须形成同源二聚体或与Fos蛋白家族成员形成异源二聚体组成转录因子AP-1。Fos家族成员不能形成同源二聚体，只能和Jun家族成员形成异源二聚体^[30]^。本研究结果表明，c-Fos在45.9%的非小细胞肺癌组织中阳性表达，而且其表达水平与COX-2和NFAT3表达水平都具有相关性。虽然在表达水平上c-Jun与COX-2和NFAT3都具有相关性，但在本研究159例肺癌组织中表达率只有14.5%。这提示，在非小细胞肺癌肺癌中c-Fos可能通过和Jun-B或Jun-D等蛋白结合组成AP-1，与NFAT3共同激活COX-2的转录，c-Jun可能在其中不起主要作用。COX-2在肺癌中表达的调节机制及其转录因子还有很多未知的领域，本研究的结果为深入进行这方面的研究提供了线索，有助于深入认识肺癌发生发展的分子改变。
